# Detection of *Mycobacterium* *avium* subspecies *paratuberculosis* infection in two different camel species by conventional and molecular techniques 

**Published:** 2015-12-15

**Authors:** Masoud Haghkhah, Abdollah Derakhshandeh, Reza Jamshidi, Asghar Moghiseh, Negar Karimaghaei, Mohammad Ayaseh, Mohsen Mostafaei

**Affiliations:** 1 *Department of Pathobiology, School of Veterinary Medicine, Shiraz University, Shiraz, Iran;*; 2 *Department of Clinical Sciences, School of Veterinary Medicine, University of Semnan, Semnan, Iran; *; 3 *Department of Clinical Sciences, School of Veterinary Medicine, Shiraz University, Shiraz, Iran; *; 4 *DVM Students, School of Veterinary Medicine, Shiraz University, Shiraz, Iran; *; 5 * Agriculture Research Centre, Ardebil, Iran.*

**Keywords:** Acid fast staining, Iranian Camel, *Mycobacterium avium*, Paratuberculosis, PCR

## Abstract

Paratuberculosis (John’s disease) is infectious and chronically progressive granulomatous disease which affects domestic and wild ruminants. The causative agent is *Mycobacterium avium paratuberculosis* (MAP)*,* a slow growing mycobactin dependent acid-fast bacillus. We investigated the detection and frequency of MAP in apparently healthy dromedary and Bactrian camels by insertion sequence 900 (IS900) polymerase chain reaction (PCR) and acid fast staining of fecal samples in Iran. Acid fast staining results showed that 6/50 (12.0%) samples of dromedary camels and 4/26 (15.3%) samples of Bactrian camels were suspected to MAP. Although the percentage of positivity for PCR assay of fecal dromedary camel was 8.0%, no bands corresponding to MAP detect in all samples of Bactrian camels. In conclusion, Although the incidence of MAP infection was low, further studies should be conducted to get more information on MAP infection in camel population, especially in areas where camels are close to other ruminants such as dairy cow, sheep and goat.

## Introduction

*Mycobacterium avium *subspecies *paratuberculosis *(MAP) causes Johne's disease (JD), also called para-tuberculosis, in domestic and wild ruminants throughout the world which estimated economic losses are over $250 per cow annually in highly infected dairy cattle herds,^1^^,^^2^ because of severe loss of slaughter weight and reduced milk production.^3^^,^^4^ Apart from its outbreak in cattle, sheep, goats and deer, paratuberculosis has also been diagnosed in a wide range of other free-ranging and domesticated ruminants including camels, llamas and alpacas.^5^^-^^7^

Dromedary camel (*Camelus dromedarius*) and Bactrian camel (*Camelus bactrianus*) are valuable domestic animals in tropical and subtropical areas used for their meat, milk and wool. Camel milk and meat are considered as an important source of protein for wide range of population.^8^

Paratuberculosis affects camels worldwide causing characteristic clinical manifestation of severe diarrhea resulting in death.^9^ The disease in camels may have a more rapid course than in cattle, with death occurrence after 4 to 6 weeks of onset of illness.^10^ In Bactrian camels, the disease was most severe in 3 to 5 year old animals.^7^

Paratuberculosis has been reported widely in many populations of camels in Asia, Middle East, Africa and the former Soviet Union.^7^^,^^11^^,^^12^ Paratuberculosis in Iranian camels is poorly documented in the literature even though the disease has been detected in some dairy cattle herds.^13^^-^^17^ To the best of our knowledge there has not been any published report of JD in Iranian camels using molecular approaches. 

To reduce the infection rate in a herd, the test and culling strategy of JD control programs require sensitive and specific diagnostic techniques. Fecal culture is considered as the gold standard for the diagnosis of MAP infected animals but requires 12 to 16 weeks;^18^ therefore the development of a rapid, sensitive and specific diagnostic method for the detection of MAP is essential in the control of Johne’s disease in economically important animals.^19^ The insertion sequence 900 (IS900) element is an insertion sequence considered to be a MAP-specific gene with 15 to 20 copies per genome and is a target for rapid detection of MAP by PCR.^20^^,^^21^ The aim of this study was to detect MAP in apparently healthy dromedary and Bactrian camels by IS900 PCR and acid fast staining of fecal samples in Iran. 

## Materials and Methods

**Collection of samples.** A total of 76 fecal (50 dromedary camel and 26 Bactrian camel) samples from apparently healthy camels with different ages were obtained during six months from Semnan and Ardebil provinces (Iran). Approximately 5 g of feces were collected from each animal with a gloved hand directly from the rectum and placed in a sterile, leak proof container. The fecal samples were stored at 4 ˚C until they were transported to the laboratory. The unprocessed fecal samples were preserved at – 20 ˚C for further studies.

**Ziehl-Neelsen acid fast staining.** For Ziehl-Neelsen staining, fecal smears were stained with carbol fuchsin Ziehl-Neelsen acid-fast stain. The smears were washed for 2 min in tap water, decolorized in two brief washes of acid alcohol (1% hydrochloric acid in 70% ethanol), washed for 2 min in tap water, and briefly counterstained with methylene blue.^22^ All smears were examined by three specialists, with multiple fields being evaluated. 

**DNA extraction.** According to Stabel *et al.*, fecal samples (1 g) were diluted in 9 mL of 1× Tris-ethylene-diamine tetra-acetic acid (EDTA) buffer (10 mM Tris-HCl, 1 mM EDTA; pH 7.6) in 15-mL polypropylene conical tubes. Samples were vortexed for 5 sec and allowed to settle for 2 min and then vortexed again. Samples were centrifuged at 200* g *for 30 sec, and the supernatants from each sample were transferred to new 15-mL conical tubes. The supernatants were then diluted 1:10, 1:100, or 1: 1,000 in 1× TE (Tris EDTA) buffer. One milliliter of each dilution was placed into 1.5 mL sterile, DNase, RNase-free Eppendorf tubes (Eppendorf, Hamburg, Germany) and centrifuged at 13,000 g for 2 min. Supernatants were discarded and pellets washed 2 times with 1 mL of 1 × TE buffer. Pellets were resuspended in 500 μL of 1 × TE buffer and placed in a heating block at 100 ˚C for 10 min. A negative control containing 1 × TE buffer was included in the heating block as a sentinel for DNA cross-contamination during sample processing. After cooling to room temperature, 4 μL of RNAase (500 μg mL^-1^) was added to each sample. Samples were stored at – 20 ˚C until PCR analyses were performed. ^23^

**Polymerase chain reaction.** IS900 PCR was performed as described by Corti and Stephan with minor modification.^24^ Resuspended DNA (5 μL) was used as the template for IS900 PCR. The primers P90, 5^'^-GAA GGG TGT TCG GGG CCG TCG CTT AGG-3^'^ and P91, 5^'^- GGC GTT GAG GTC GAT CGC CCA CGT GAC-3^'^ were used for amplification cycles that generated a 413 bp product. The PCR mix consisted of 25 μL reaction volume containing 20 pmol of each primer, 5 mM MgCl_2_ (CinnaGen Co., Tehran, Iran), 40 μM 2 deoxyribonucleoside- 5-triphosphate (dNTP; Cinna-Gen), 0.5 U Taq polymerase (CinnaGen) in 1× reaction buffer (CinnaGen). PCR program was as follows: 94˚C for 5 min (1 cycle); 94˚C for 1 min, 59˚C for 1 min, 72 ˚C for 2 min (30 cycles); 72 ˚C for 7 min (1 cycle). PCR products were visualized by gel electrophoresis on 1% agarose gel. *Mycobacterium avium* subspecies *paratuberculosis *ATTC 43105 was used as positive control for each PCR round.

**Statistical analysis.** Prevalence of positive and negative results for Ziehl-Neelsen acid fast staining and IS900 PCR were primarily analyzed by contingency tables. All proportions were compared using the Fisher’s exact test and a *p*-value less than 0.05 was considered significant by SPSS (version 16.0, SPSS Inc., Chicago, USA).

## Results

**Ziehl-Neelsen Staining analysis of the fecal smears.** Acid fast staining results showed that 6/50 (12.0%) samples of dromedary camels and 4/26 (15.3%) samples of Bactrian camels were suspected to MAP. But there was no significant difference (*p* = 0.7) between the prevalence of positive acid fast staining results in two groups of dromedary and Bactrian camels. 

**Fig. 1:**
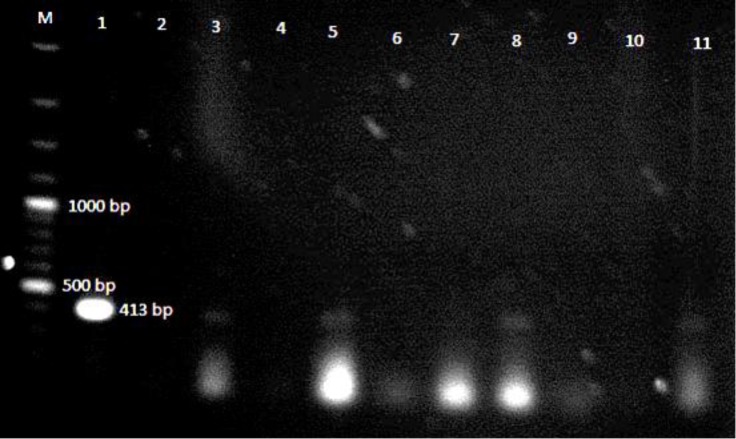
Gel electrophoresis of IS900 PCR products from fecal samples of two different camel species. Lane M: 100 bp DNA ladder. Lane 1: Positive control MAP 43105, Lane 2: Negative control, Lanes 3, 5, 8 and 11: PCR product of 413 bp from fecal samples. Lanes 4, 6, 7, 9 and 10: No product from fecal sample

**Polymerase chain reaction analysis.** A total of four dromedary faecal samples out of 50 were positive by PCR assay for Map yielding an expected PCR product of size 413 bp (Fig. 1). The percentage of positivity for PCR assay of fecal dromedary camel was 8.0%. Although expected PCR bands size of MAP revealed in fecal samples of dromedary camels, there were not bands corresponding to MAP detection in all samples of Bactrian camels. No significant difference (*p* = 0.2) was found between the percentage of positivity for IS900 PCR assay in two groups of dromedary and Bactrian camels.

## Discussion

Johne’s disease is an infectious disease of cattle and other ruminants, caused by *Mycobacterium avium* subspecies *paratuberculosis* (MAP).^6^^,^^7^ Diagnosis of paratuberculosis is difficult because of the fastidious growth pattern of the microorganism and the different host immune responses invoked during subclinical and clinical stages of infection. Traditionally, fecal culture for MAP is considered as the gold standard for diagnosis.

However, fecal culture is time-consuming and detects only 38.0 to 50.0% of infected animals.^23^ Serological tests such as enzyme-linked immunosorbent assay (ELISA) are even less sensitive than fecal culture, particularly in apparently healthy or sub-clinically infected animals.^8^ Use of nucleic acid probes combined with the PCR for detection of MAP in fecal samples have vastly improved in recent years, leading to an increased sensitivity in detecting of low shedders, including a detection level of one colony-forming unit(s) per gram of feces. This is done by amplifying the IS900 gene sequence which is the most reliable way of detecting cows shedding low levels of MAP in their feces.^23^^,^^25^^,^^26^

Use of PCR in contrast to culture and serological tests, allowed to detect nonviable as well as viable micro-organisms and would be a more sensitive detection method. Therefore, in comparison with serological or culture methods, detection of MAP directly from bulk-tank milk or feces by IS900 PCR could be considered a valuable test for the estimation of herd-level prevalence.^27^

 The MAP has been detected in dairy herds throughout Iran.^13^^-^^15^ A recent work in dairy cow herds in Fars province (southern Iran) showed a herd-level prevalence of 11.0% based on IS900 nested PCR on bulk-tank milk samples.^28^ Another study done by nested PCR also showed that, 5 out of 90 samples (5.6%) were positive for MAP. However, the frequency of infection was diverse in different regions ranging from 4.2% to 7.7%.^26^ Khaled *et al*., confirmed infection with MAP by PCR by targeting the IS900 gene. They extracted DNA from lymph node and liver samples of the five suspected camels resulted in amplification of a 229-bp PCR product which is the specific product of *M. paratuberculosis*–IS900.^25^

Alhebabi and Alluwaimi reconfirmed the spread of MAP infection in the Saudi camel herds and concluded the ruminant ELISA was proved useful for the screening of the MAP infection in camel and could rule out the need for the species specific ELISA test. They have shown their feasibility as robust diagnostic tool for screening of John’s disease in camel. They also proved that PCR was more practical than ELISA in detecting MAP.^8^ In this study, according to previous study,^23^ we used a rapid and simple DNA extraction method for the detection of MAP in fecal samples which were taken from two different camel species. Acid fast staining of suspected tissues is rapid and requires little optimization. However, Ziehl-Neelsen staining has been reported to falsely identify *Nocardia *and *Corynebacteria* and cannot differentiate among the various mycobacterial species. Previous reports have determined the sensitivity of Ziehl-Neelsen to be 36.4%.^29^

A total of 4 out of 50 dromedary camel samples were positive in fecal samples by PCR. The percentage of positivity for PCR detection in this study was 8.0%. This result is in agreement with that of Alhebabi and Alluwaimi who reported only 97 positive samples out of 310 tested samples by PCR in dromedary camels.^8^ Although the outbreak of MAP infection in Bactrian camels (*Camelus bactrianus*) was 15.3% based on acid fast staining, acid fast staining could not completely predicative. The results showed no bands on fecal samples by IS900 PCR, so it could be concluded that the genus *Camelus bactrianus* from our region are may be free of infection but further research is required. The study has confirmed the spread of MAP infection in the dromedary camel in Iran. But there is no significant association (*p* > 0.05) between the camel species and the prevalence of MAP infection by either acid fast staining or IS900 PCR methods.

In the present study, IS900 PCR assay proved to be a more sensitive and reliable than acid fast staining for the detection of MAP in faecal samples of camels since the PCR assay was able to detect significantly more positive cases than acid fast staining. This study suggests that IS900-PCR-based detection of MAP could be used as a potential diagnostic tool for rapid and effective Johne’s disease surveillance in camels.

To the best of our knowledge, this study is the first description of a work of this kind performed in Iran. Although the incidence of MAP infection was low, further studies should be conducted to get more information on MAP infection in camel population, especially in areas where camels are close to other ruminants such as dairy cows, sheep and goats. 
